# Effects of Ripasudil on Open-Angle Glaucoma after Circumferential Suture Trabeculotomy Ab Interno

**DOI:** 10.3390/jcm10030401

**Published:** 2021-01-21

**Authors:** Tomoki Sato, Takahiro Kawaji

**Affiliations:** Sato Eye and Internal Medicine Clinic, 4160-270 Arao, Arao City, Kumamoto 860-0041, Japan; zxsato@gmail.com

**Keywords:** ripasudil, suture trabeculotomy ab interno, Schlemm’s canal incision, aqueous outflow, open-angle glaucoma

## Abstract

We evaluated the effects of ripasudil on the distal aqueous outflow tract in patients with open-angle glaucoma (OAG) who underwent a 360° suture trabeculotomy ab interno followed by ripasudil treatment beginning 1 month postoperatively. We compared 27 of these patients, by using propensity score analysis, with 27 patients in a matched control group who had no ripasudil treatment. We assessed the changes in the mean intraocular pressure (IOP) and the relationship between the IOP changes and background factors. All eyes had a complete 360° Schlemm’s canal incision and phacoemulsification. The mean IOP at 1 and 3 months after ripasudil administration were significantly reduced by −1.7 ± 1.9 mmHg (*p* < 0.0001) and −1.3 ± 2.3 mmHg (*p* = 0.0081) in the ripasudil group, respectively, but IOP in the control group was not significantly reduced. The IOP reduction was significantly associated with the IOP before ripasudil treatment (*p* < 0.001). In conclusion, the use of ripasudil for patients with OAG after circumferential incision of the Schlemm’s canal produced significant IOP reductions. Ripasudil may affect the distal outflow tract, thereby leading to the IOP reduction.

## 1. Introduction

Glaucoma is an ocular condition that manifests functional and structural abnormalities of the optic nerve related to retinal ganglion cell death [1,2]. Elevated intraocular pressure (IOP) is known to be a main risk factor for the onset and progression of glaucoma, so lowering the IOP has been believed to be the main treatment strategy to prevent vision loss in patients with glaucomatous eyes [3,4]. Aqueous humor drains through the conventional outflow pathway via the trabecular meshwork and the uveoscleral pathway through the ciliary body to the suprachoroidal space. This trabecular meshwork structure is thought to be responsible for the highest resistance to aqueous outflow [5,6,7]. However, several experiments and surgical outcomes have suggested that one-third to one-half of the total outflow resistance is located distal to Schlemm’s canal [8,9,10]. A number of medications, such as prostaglandin analogs, beta-blockers, carbonic anhydrase inhibitors, and α2-agonists, are used to lower IOP levels in glaucomatous eyes; however, these drugs affect the uveoscleral pathway or aqueous humor production as opposed to the conventional outflow pathway.

In addition to these IOP-lowering drugs, inhibitors of Rho-associated protein kinase (ROCK) have been developed to reduce IOP levels in animal and human eyes [11,12]. The IOP-lowering effects of ROCK inhibitors have induced alterations in cell shape, contraction, motility, attachment, and extracellular matrix production in the trabecular meshwork and Schlemm’s canal endothelial cells, which have resulted in improvements in the conventional aqueous outflow [12]. Certain ROCK inhibitors, including ripasudil (Glanatec ophthalmic solution 0.4%; Kowa Company, Ltd., Nagoya, Aichi, Japan) and netarsudil (Rhopressa ophthalmic solution 0.02%; Aerie Pharmaceuticals, Bedminster, NJ, USA), are now available in the market and are used in clinical practice in a number of countries [13,14]. In animal models, enucleated human eyes and living human eyes, netarsudil increased outflow facility by expanding the juxtacanalicular trabecular meshwork and by dilating episcleral veins [15,16,17,18,19,20]. However, the effects of ripasudil, one of the ROCK inhibitors, on the distal outflow pathway in living human subjects have not yet been studied.

We have seen several reports concerning the clinical outcomes of 360° suture trabeculotomy ab interno used for open-angle glaucoma (OAG) [21,22]. This procedure can relieve the resistance to aqueous humor outflow by using an incision of the trabecular meshwork and the inner layer of Schlemm’s canal. Therefore, the function of the distal outflow tract can be evaluated after the circumferential incision of Schlemm’s canal via this procedure. In our previous studies, we showed that the IOP was reduced and fluctuated within 1 month after the surgery, and then, from 1 month postoperatively, the IOP stabilized in most cases [21,22]. Therefore, in the present study, we evaluated the effects of ripasudil on the distal aqueous outflow tract in patients with OAG who underwent the circumferential incision of Schlemm’s canal, followed by ripasudil treatment beginning 1 month postoperatively. We then compared those patients with a matched control group, i.e., patients who had no ripasudil treatment postoperatively. We utilized a quasi-randomized propensity score analysis to minimize the influence of the confounding factors.

## 2. Materials and Methods

### 2.1. Study Design and Patients

This analysis was a retrospective, single-center, observational, comparative clinical study performed at Sato Eye and Internal Medicine Clinic (Arao City, Kumamoto, Japan) between 2014 and 2019. The study protocol adhered to the principles of the Declaration of Helsinki and was approved by the Institutional Review Board and the Ethics Committee at Sato Eye and Internal Medicine Clinic. Informed consent was obtained from all patients.

Patients were considered for admission into the study if they had OAG together with a visually significant cataract. We reviewed the medical charts of 42 consecutive patients who underwent a 360° suture trabeculotomy ab interno with phacoemulsification, and 1 month later these patients started to receive ripasudil between 2018 and 2019 (ripasudil group); alternatively, we reviewed 52 patients who underwent a 360° suture trabeculotomy ab interno with phacoemulsification without additional treatment after surgery between 2014 and 2017 (control group). Patients were followed up for at least 3 months after the start of ripasudil treatment. We did not include patients who had suture trabeculotomy ab interno alone because of the small number of these patients.

Study patients had primary open-angle glaucoma (POAG), normal-tension glaucoma, or exfoliation glaucoma. Glaucoma surgeries were performed both in medically uncontrolled eyes and in medically well-controlled eyes to reduce the medication burden. A cataract was said to be visually significant if a patient reported a glare or halos and had a best-corrected visual acuity of 20/20 or worse. If subjects had any of the following, they were excluded from this study: other types of glaucoma; a history of incisional glaucoma surgery; use of additional anti-glaucoma agents except ripasudil or surgery during a postoperative 3-month period; and being under 20 years of age at the time of the surgery. One surgeon (T.S.) performed all operations; he used the surgical procedure described here [21].

The primary outcome measure was the mean IOP change at each visit. We also assessed the relationship between the change in IOP and the background factors in the ripasudil group.

### 2.2. Surgical Technique

The suture trabeculotomy ab interno procedure used here was previously described in full [21]; in the present study, a modified technique for Schlemm’s canal incision was used. In brief, a 1.7 mm temporal corneal incision was made, and the Schlemm’s canal on the nasal side was incised at 15° by utilizing a microhook needle (HS-2167; Handaya, Tokyo, Japan) instead of the trabectome (NeoMedix, Tustin, CA, USA), as used in the original paper [21]. A standard phacoemulsification with intraocular lens implantation in the capsular bag was performed through the same or a new upper corneal incision (depending on the easier procedure in each case), with the phacoemulsification with lens implantation being performed after the suture trabeculotomy ab interno. At the end of operation, we used iCare (M.E. Technica, Tokyo, Japan) to determine that no wound leakage existed and that the IOP value in the supine position was 18 mmHg or more. Postoperative drug therapy consisted of a 2-week regimen of moxifloxacin hydrochloride, 0.1% betamethasone, and 2% pilocarpine.

### 2.3. Data Collection and Statistical Analysis

All potential subjects were screened for eligibility by using the following methods: slit-lamp biomicroscopy, indirect ophthalmoscopy, manifest refraction, measurement of IOP, and best-corrected visual acuity assessment (via the conventional Landolt ring chart). The surgeons or other ophthalmologists used a Goldmann tonometer to measure all IOP values. The mean of the 3 most recent measurements, acquired on separate days within 1 month before surgery, was utilized to determine the baseline IOP. An Auto Ref/Keratometer (ARK-530A; Nidek, Tokyo, Japan) was used to measure the manifest refraction; decimal visual acuity values were changed to the logarithm of the minimum angle of resolution.

All patients were evaluated on days 1, 2, and 3 after surgery, and then every 1–2 weeks for 1 month, after which ripasudil was administered. After the start of ripasudil treatment, patients were examined at 1, 2, and 3 months. The IOP was measured at every visit.

The JMP statistical package (version 14; SAS Institute Inc., Cary, NC, USA) was used to analyze all data. Cases were matched via propensity score analysis by using a genetic algorithm based on age, sex, type of glaucoma, preoperative IOP and medications, 1-month postoperative IOP, axial length, and visual field. Continuous data were expressed as means ± standard deviation, and changes in continuous variables were assessed by using the paired *t*-test. Multiple regression models were utilized to analyze associations between IOP changes at 3 months after ripasudil treatment and background factors. The statistical significance value was set at *p* < 0.05.

## 3. Results

### 3.1. Disposition and Characteristics of Patients

From among 40 patients in the ripasudil group and 54 patients in the control group, 54 patients were included in this study—27 patients in a ripasudil group and 27 patients in a control group. The propensity score analysis revealed no significant difference in baseline characteristics between the two groups (Table 1). Suture trabeculotomy ab interno combined with phacoemulsification was performed in all eyes, and all eyes had a complete 360° Schlemm’s canal incision.

### 3.2. Efficacy

**Figure 1:**
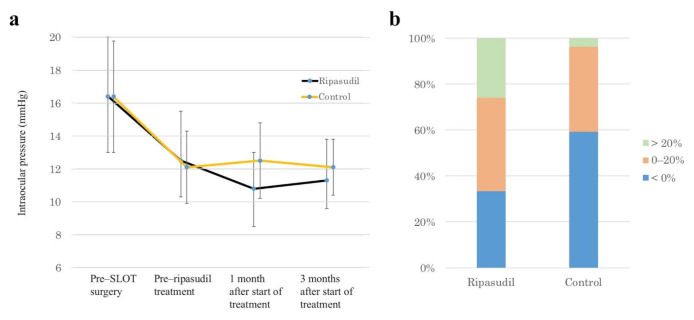
IOP–lowering effects of ripasudil. (**a**) Time–dependent changes in IOP at different time points: pre–SLOT surgery, pre–ripasudil treatment (i.e., 1 month post-operation), and 1 month and 3 months after the start of ripasudil treatment. (**b**) Stacked bar chart of IOP changes at 3 months after the start of ripasudil treatment. SLOT suture trabeculotomy ab interno.

As Figure 1a shows, the mean IOP value was significantly reduced from 16.4 ± 3.6 mmHg to 12.5 ± 3.0 mmHg (*p* < 0.0001) at 1 month post-operation in the ripasudil group and from 16.4 ± 3.4 mmHg to 12.1 ± 2.2 mmHg (*p* < 0.0001) at 1 month post-operation in the control group; ripasudil treatment was then started in the ripasudil group. The mean IOP values at 1 and 3 months after the start of ripasudil treatment were significantly reduced by −1.7 ± 1.9 mmHg (*p* < 0.0001) and −1.3 ± 2.3 mmHg (*p* = 0.0081) in the ripasudil group, respectively, and there was no significant difference between at 1 and 3 months IOP (*p* = 0.3013). At these same time points, however, the IOP reductions were not significant in the control group: 0.4 ± 2.2 mmHg (*p* = 0.8494) and 0.1 ± 2.0 mmHg (*p* = 0.9251), respectively. The IOP values at 1 month (*p* = 0.0003) and 3 months (*p* = 0.0317) after ripasudil treatment were significantly different between the ripasudil group and the control group. At both 1 and 3 months after ripasudil treatment, no IOP reduction was seen in 33% of the ripasudil group; at that same time point, 60% of the control group showed no IOP reduction (Figure 1b).

Multiple regression analysis of data for the ripasudil group demonstrated that the IOP reduction at 3 months was significantly associated with the IOP value before ripasudil treatment, but not with age, visual field, preoperative medications, or IOP value before surgery (Table 2).

## 4. Discussion

In this retrospective study, we used propensity score analysis to investigate the effects of ripasudil treatment on patients with OAG after 360° suture trabeculotomy ab interno, in whom the aqueous outflow resistance caused by the trabecular meshwork should be eliminated, and we found a significant IOP reduction. This finding suggested that ripasudil may affect the distal outflow tract, with the consequence being IOP reduction.

Although IOP elevation was long thought to be caused by outflow resistance at the trabecular meshwork, which blocks the eye’s drainage system, data from clinical studies of trabecular meshwork-targeted minimally invasive glaucoma surgery (MIGS) showed that the MIGS failed to lower the IOP to the pressure level in the recipient episcleral veins [23,24]. Previous studies in enucleated human eyes suggested that 25–50% of total outflow resistance is located at the conventional outflow pathway distal to Schlemm’s canal [8,9]. Research utilizing multimodal two-photon-deep tissue imaging and three-dimensional analysis demonstrated that the distal aqueous drainage tract wall can contract and aid the dynamic alteration of the drainage tract, similarly to the function of blood vessel tone in blood flow regulation [25]. Another study showed that the regulation of distal vessels via the physiological vasoregulators endothelin-1 and nitric oxide dramatically affected outflow resistance in both humans and pigs [26]. Thus, distal vessels probably play an important role in regulating outflow resistance, especially after MIGS and perhaps in the pathophysiology of glaucomatous eyes.

Several reports showed the effects of netarsudil on the structure and function of the distal outflow tract; the dilation of outflow tract vessels together with a corresponding pressure reduction was observed in mouse [17], rabbit [16], porcine [19] and ennucleated human eye models [18], and living human eye [20]. However, the effects of ROCK inhibitors on the distal outflow tract in living human subjects has not been fully studied. We demonstrated here that glaucomatous eyes that had a circumferential incision of the trabecular meshwork via 360° suture trabeculotomy demonstrated a significant IOP reduction after ripasudil treatment. We started ripasudil administration 1 month post-operation, because we had found that the IOP fluctuated for 1 month after suture trabeculotomy and then, from 1 month postoperatively, the IOP stabilized in most cases [21,22]. In this study, we included a control group and used propensity score analysis to evaluate whether the IOP actually stabilized from 1 month postoperatively. Our result clearly showed that the IOP stabilized from 1 month postoperatively in the control group; therefore, a significant IOP reduction after the administration of ripasudil may be caused by the effect of ripasudil, and may not be the natural postoperative course. In addition, we excluded a patient who also required anti-glaucoma medication other than ripasudil, because such a patient may already have a non-functional distal outflow tract. Thus, our study protocol made the evaluation of the function of an almost intact distal outflow tract possible. Multiple regression analysis showed that the IOP reduction was significantly associated with only the IOP value before ripasudil treatment. As Figure 1b shows, no IOP reduction at 3 months after the start of ripasudil treatment was observed in about one-third of the eyes. The absence of IOP reduction in these eyes may be explained by a lack of efficacy of ripasudil at lower IOP values, but the reason for this finding is unknown.

Trabecular meshwork-targeted MIGS have gained popularity for lowering the IOP, so the imaging and understanding of the distal outflow tract have increased in importance. Recent publications reported intraoperative observations of fluid waves in episcleral veins, which were correlated with good postoperative IOP reduction and/or the number of medications [27,28]. Goda et al. reported that success probabilities for trabeculotomy ab externo were 100% for patients with POAG who achieved an IOP value < 21 mmHg via preoperative ripasudil administration [29]. This result suggested that whether ripasudil is effective or not before trabeculotomy may help predict to what degree the trabecular meshwork, as well as possibly the distal outflow tract, is functional, and may be used as a trabeculotomy outcome marker in patients with POAG. In this study, this hypothesis could not be evaluated because of the small number of patients who received ripasudil before surgery.

Recent MIGS procedures have been routinely performed in medically well-controlled eyes to reduce the medication burden, and as an early intervention in eyes requiring IOP reduction without medications or laser treatments at the time of cataract surgery [30,31]. Thus, such cases were included in the present study and had relatively low baseline IOP and 1-month postoperative IOP. Our data showed that the mean 1-month postoperative IOP was about 12 mmHg; no medication is usually used for eyes having such a low IOP in clinical settings. We had thought to investigate whether ripasudil could affect the distal outflow tract in living humans; on the basis of this study, however, we cannot determine whether ripasudil is clinically useful from the point of view of glaucoma management. Additional investigations of eyes with higher IOP values and/or more severe stages of glaucoma are needed to ascertain whether ripasudil should be used after circumferential incision of the Schlemm’s canal, given the expected effect of ripasudil on the distal outflow tract.

The present study has certain limitations. This study is retrospective. Additionally, although we used propensity score matching to overcome the study limitations, propensity score analysis, unlike prospective randomized clinical trials, cannot randomize unknown confounding factors and loses certain samples through 1:1 matching analysis. This study also includes the possibility of selection bias because of its single institution-based nature with a small sample size. In addition, all eyes underwent suture trabeculotomy ab interno plus phacoemulsification. The eyes that underwent only suture trabeculotomy ab interno (i.e., without phacoemulsification) may have demonstrated a more dramatic result, but the number of eyes was small. Phacoemulsification may have further limited the effect of ripasudil and may have affected the IOP changes after surgery or after ripasudil administration. Therefore, eyes that underwent suture trabeculotomy ab interno with phacoemulsification, but without ripasudil treatments, were included as a control group.

In conclusion, the ripasudil treatment of patients with OAG after 360° suture trabeculotomy ab interno led to a significant reduction in IOP. Ripasudil may affect the distal outflow tract and result in an IOP reduction in living human subjects.

## Figures and Tables

**Table 1:** Baseline characteristics of patients.

Characteristic	Ripasudil	Control	*p*-Value
No. of patients	27	27	
Age, years, mean ± SD (range)	79.7 ± 6.8 (63–88)	78.5 ± 7.6 (64–89)	0.548
Male/female, *n*	15/12	11/16	0.414
Right/left, *n*	11/16	13/14	0.352
IOP, mmHg, mean ± SD (range)			
Pre-SLOT surgery	16.4 ± 3.6 (11–26)	16.4 ± 3.4 (11–25)	0.785
Pre-ripasudil treatment	12.5 ± 3.0 (6–21)	12.1 ± 2.2 (7–16)	0.775
No. of preoperative medications, mean ± SD (range)	1.9 ± 1.3 (0–4)	1.4 ± 1.5 (0–4)	0.525
BCVA, logMAR, mean ± SD (range)	0.26 ± 0.21 (0–0.82)	0.35 ± 0.22 (0–1.10)	0.295
Visual field, MD (dB), mean ± SD (range)	−8.6 ± 5.4 (−18.4–−0.2)	−9.7 ± 8.1 (−27.9–−1.6)	0.106
Axial length, mm, mean ± SD (range)	23.7 ± 0.8 (22.5–25.8)	24.2 ± 1.5 (21.3–27.4)	0.569
Type of glaucoma, *n* (%)			0.211
POAG	10	13	
NTG	10	11	
XFG	7	3	

IOP, intraocular pressure; SLOT, suture trabeculotomy ab interno; BCVA, best-corrected visual acuity; logMAR, logarithm of minimum angle of resolution; MD, mean deviation; POAG, primary open-angle glaucoma; NTG, normal-tension glaucoma; SD, standard deviation; XFG, exfoliation glaucoma.

**Table 2:** Relationship between IOP changes at 3 months after the start of ripasudil treatment and background factors, as determined by multiple regression analysis.

Background Factor	T-Value	*p*-Value
IOP (mmHg) before surgery	1.67	0.097
IOP (mmHg) before ripasudil treatment	−7.28	<0.001
Medication before surgery	1.99	0.050
Age (years)	0.38	0.702
Visual field (mean deviation, dB)	−0.39	0.699

IOP intraocular pressure.

## Data Availability

The data analyzed in this study are available from the corresponding author on reasonable request.
